# Patients with functional gastrointestinal disorders—importance of communication between physician and patient assessed in a cross-sectional cohort study

**DOI:** 10.3389/fpsyt.2023.1252268

**Published:** 2023-08-31

**Authors:** Miriam Goebel-Stengel, Ute Paulsen, Petra Bennerscheidt, Stephan Zipfel, Andreas Stengel

**Affiliations:** ^1^Department of Internal Medicine, Psychosomatic Medicine and Psychotherapy, University Hospital Tübingen, Tübingen, Germany; ^2^Department of Internal Medicine, Helios Clinic, Rottweil, Germany; ^3^Dr. Willmar Schwabe GmbH & Co. KG, Karlsruhe, Germany; ^4^Charité Center for Internal Medicine and Dermatology, Department for Psychosomatic Medicine, Charité—Universitätsmedizin Berlin, Corporate Member of Freie Universität Berlin, Humboldt-Universität zu Berlin and Berlin Institute of Health, Berlin, Germany

**Keywords:** functional dyspepsia, general practitioner, internal medicine, irritable bowel syndrome, somatoform

## Abstract

Functional gastrointestinal disorders are frequent diseases often associated with a pronounced burden reflected in a greatly reduced quality of life. Patients are seeking medical help but may be perceived as demanding and challenging. For successful diagnosis and treatment of these patients, a good doctor-patient communication is key. However, so far, only few studies focus on the physicians’ perspective of the doctor-patient communication. The present study cross-sectionally investigated 520 physicians using the validated difficult doctor-patient relationship questionnaire and the treatment satisfaction questionnaire from the physician’s perspective along with several *ad hoc* questions. Data from 5,354 physician-patient conversations (one conversation per patient) was included. Physicians participating in this study mostly suspected stress-related burdens as the cause of functional gastrointestinal disorders (65.4%), while patients rather suspected food (55.4%) or other somatic causes (43.6%). The physician-patient relationship was rated just below the threshold for difficult interactions (cut-off ≥30, mean ± SD in the current sample: 28.6 ± 9.6) with 49.1% of physicians reaching a score of ≥30. Although physicians overall felt confident in the doctor-patient communication even in difficult conversations (61.9%), only 33.1% reported to have enough time for these patients and only 5.6% felt sufficiently compensated for discussions with patients with functional gastrointestinal disorders. Therefore, education of physicians on functional gastrointestinal disorders, training of physicians in physician-patient communication as well as an improved reimbursement of speaking medicine should help to further improve care for these patients and also treatment satisfaction on both the side of the patients as well as the physicians.

## Introduction

Functional gastrointestinal disorders (FGID), recently also known as disorders of the brain gut interaction, such as functional dyspepsia (FD) (1) and irritable bowel syndrome (IBS) (2) are common disorders affecting about 10% of the adult population. These disorders cannot be explained purely by somatic findings but have multifactorial causes summarized in the biopsychosocial model (1, 2). They are associated with a severely impaired quality of life (1, 2). For coping with the disease, the doctor-patient relationship is of crucial importance, which, however, often leads to inefficient interaction: in search of help, the patient visits the physician, who searches in vain for an organic cause, leading to referrals, repeated examinations, (unnecessary) drug treatments, and even surgical interventions (3). When these measures do not help, the patient becomes dissatisfied, pessimistic, and anxious, and focuses more on the symptoms, which in turn leads to an increase in symptoms and another visit to the/another doctor, setting off a vicious cycle (3). Patients with functional gastrointestinal disorders are often perceived as difficult by treating physicians (4). About one-third of physicians reported frustration in treating patients with IBS (4). However, data are scarce. In this study, empirical data were collected from general practitioners about their communication with patients with functional gastrointestinal disorders to address the following research questions: do doctors and patients have different hypotheses on the development of the functional gastrointestinal disorder? Do physicians initiate different measures than desired by the patients? Are interactions between doctors and patients with functional gastrointestinal disorders perceived as difficult by the physicians? Is the treatment of patients with functional gastrointestinal disorders judged as satisfactory by the physicians? How do physicians overall judge their skills in doctor-patient communication?

## Methods

The present study cross-sectionally examined physician-patient communication as assessed by primary care physicians in Germany. It was planned to recruit 785 GPs for the study, 743 (94.6%) were recruited over a six-month period in 2021, of whom 568 turned in data, and ultimately 520 were included with complete self-assessment data sets (66.2%). Data from a total of 5,428 patients (documented physician-patient conversations after consultation with a patient with a functional gastrointestinal disorder, visit related to FGID symptoms, maximum of 10/physician) were collected. Due to missing data (>1% of items) 74 assessments were excluded, so ultimately 5,354 documented physician-patient conversations were included (98.6%).

Physicians practicing as primary care physicians evaluated each of the 5,354 conversations using *ad hoc* questions as well as the validated difficult doctor-patient relationship questionnaire (DDRPQ-10, German version, 10 items, Likert scale from 1 not at all to 6 to a large extent, sum score ≥30 indicates difficult doctor-patient relationship, internal consistency of current sample 0.70) (5). The 10 items were as follows:

- How much are you looking forward to this patient’s next visit after seeing this patient today?
- How frustrating do you find this patient?
- How manipulative is this patient?
- To what extent are you frustrated by this patient’s vague complaints?
- How self-destructive is this patient?
- Do you find yourself secretly hoping that this patient will not return?
- How at ease did you feel when you were with this patient today?
- How time consuming is caring for this patient?
- How enthusiastic do you feel about caring for this patient?
- How difficult is it to communicate with this patient?

Moreover, physicians filled in the treatment satisfaction questionnaire from the physician’s perspective (11 items for current treatment, Likert scale from 0 not at all to 5 very, no sum score in use, internal consistency 0.94) (6). The respective 11 items were as follows:

- How successful do you consider your current treatment to be?
- How satisfied are you with your current treatment?
- To what extent do you think the patient felt understood by you as a physician?
- Do you think your patient felt his complaints were taken seriously?
- Have you explained to your patient in detail where his complaints may be coming from?
- Do you have the impression that he could agree with these statements?
- Did you address your patient’s ideas about the origin and treatment of the complaints?
- To what extent did you get the impression that your patient and you meant the same thing when you talked about his complaint and its treatment?
- Did you feel like you included his perspective and opinion when planning treatment?
- How responsive were you to the patient’s emotional state?
- To what extent did you also ask about stresses, for example, at work or in the family?

Lastly, physicians once completed a general self-assessment questionnaire encompassing 11 items. Before the study, physicians were instructed how to fill in the general self-assessment (once) as well as 10 questionnaires on a communication between doctor and patient with a functional gastrointestinal disorder directly after the respective communication. All ratings were performed by the physician including the suspected patient wishes.

The study was approved by the Ethics Committee of the University Hospital of Tübingen (395/2020BO). The statistical analyses including plausibility checks were performed by INPADS GmbH, Bad Dürkheim, Germany. No outliers were removed, no missing data were imputed (less than 1% of missing items were tolerated).

## Results

### Characterization of participating physicians

Most of the physicians participating in this study (*n* = 520) had an age between 50–59 (38.5%) followed by 60–69 (23.5%, Table 1). In line with these data, physicians reported practicing medicine for 10–19 (30.9%) and 20–29 years (29.8%), respectively (Table 1). Most physicians were general practitioners (74.2%) followed by doctors specialized in internal medicine (24.4%, Table 1).

**Table 1:** Characterization of participating physicians (*n* = 520).

Characteristic	Frequency in % (*n*)
*Age (years)*	
< 30	1.0 (5)
30–39	11.9 (62)
40–49	21.2 (110)
50–59	38.5 (200)
60–69	23.5 (122)
≥ 70	2.7 (14)
Missing data	1.3 (7)
*Duration of medical practice (years)*	
< 10	16.9 (88)
10–19	30.9 (160)
20–29	29.8 (155)
≥ 30	18.5 (96)
Missing data	4.0 (21)
*Specialization*	
General practice^a^	74.2 (386)
Internal medicine^a^	24.4 (127)
Other^a^	12.7 (66)
Missing data	0.8 (4)

a Multiple choice possible.

### Characterization of participating patients

The age of patients participating/being evaluated in this survey (*n* = 5,354) was evenly distributed between 18 and 70 years (Supplementary Table S1). Likewise, the duration of symptoms leading to the current consultation was evenly distributed ranging from less than 4 weeks (19.3%) to more than 5 years (10.2%, Supplementary Table S1). Most patients rated their impairment due to the complaints (Likert scale from 1 none to 6 maximum) as 4 (29.2%) or 5 (33.8%, Supplementary Table S1).

### Hypotheses on the development of the functional gastrointestinal disorder

**Figure 1:**
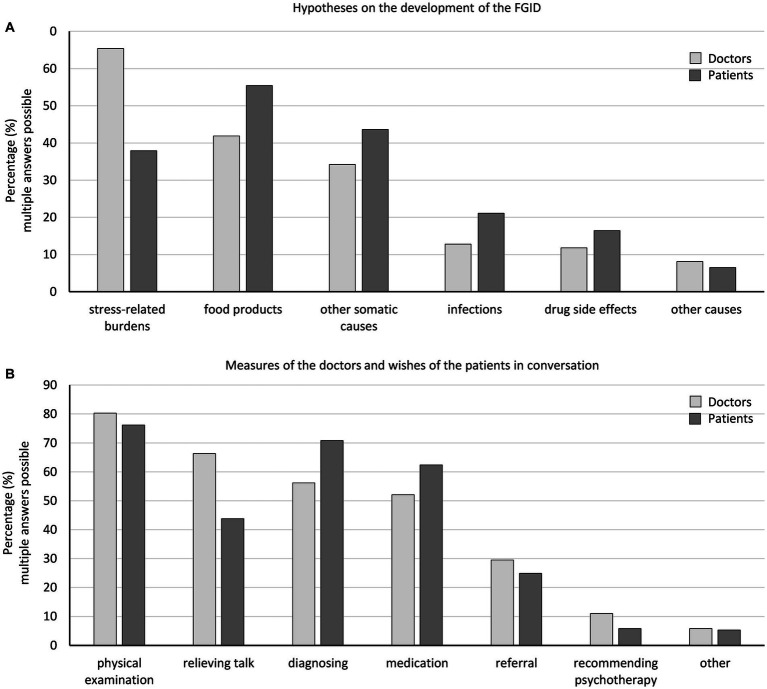
Physicians’ and patients’ hypotheses about the development of the functional gastrointestinal disorder **(A)** and physicians’ measures and patients’ wishes in the interview **(B)**.

While physicians mostly suspected stress-related burdens as the cause of functional gastrointestinal disorders (65.4%), patients who most frequently reported bloating (70.9%), pain/cramps (63.7%), bowel movement changes (52.9%), and nausea (30.6%) as bothersome (data not shown) were more likely to suspect food (55.4%) or other somatic causes (43.6%) as the main reasons (Figure 1A).

### Measures of the doctors and wishes of the patients

Patients desired (76.2%) and received a physical examination (80.3%), whereas physicians offered a relieving conversation (66.3%) more often than desired (43.8%), and patients more often expected further diagnosing (patients vs. doctors, 70.8 vs. 56.2%) and medications (62.4 vs. 52.1%, Figure 1B).

### Difficult doctor-patient relationship questionnaire

**Figure 2:**
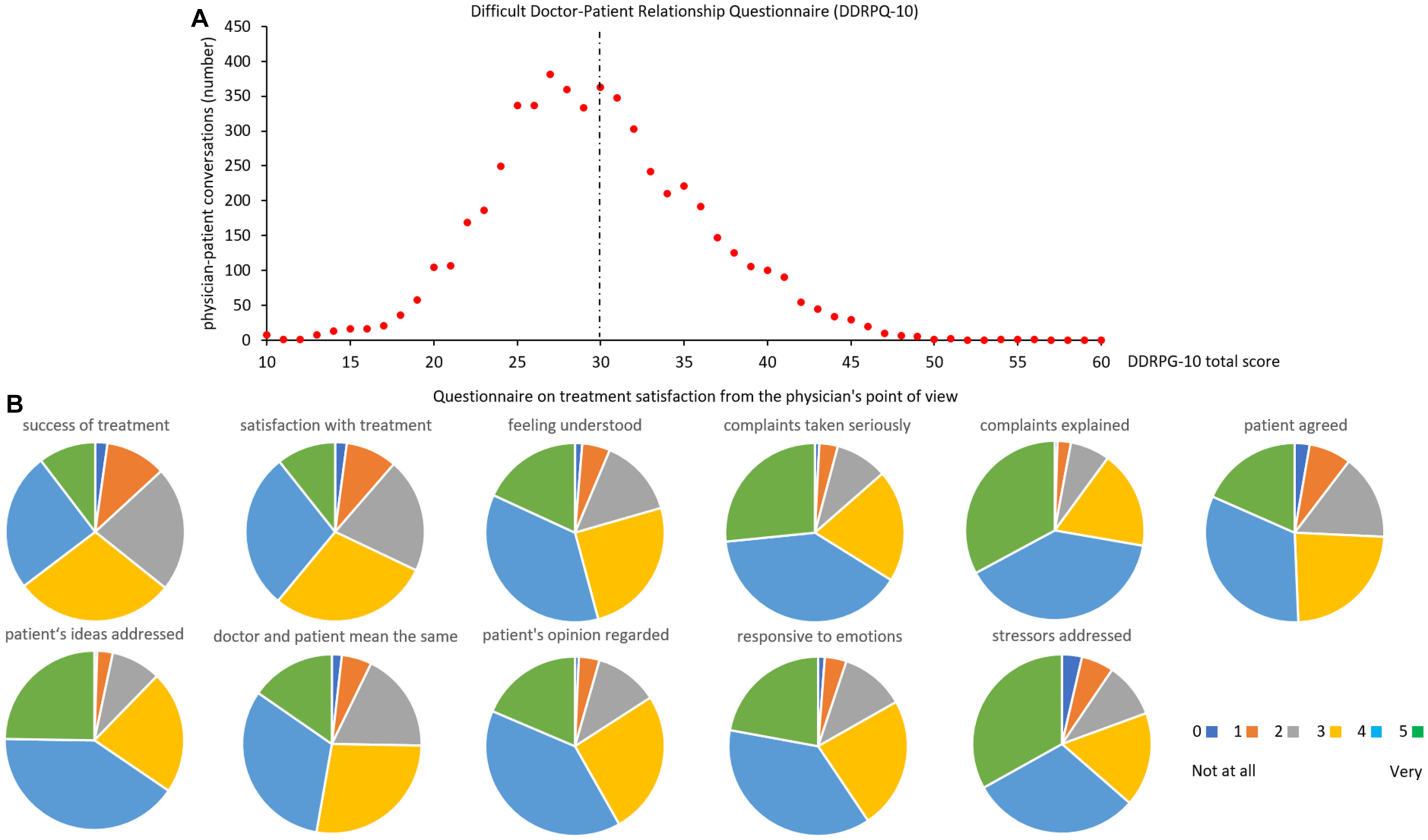
Difficult doctor-patient relationship questionnaire (DDRPQ-10, distribution of total score, **A**) and treatment satisfaction questionnaire from the doctor’s perspective (single items **B**). The dotted line in **(A)** indicates the cutoff. Abbreviations of items: success of treatment: how successful do you consider your current treatment to be?; satisfaction with treatment: how satisfied are you with your current treatment?; feeling understood: to what extent do you think the patient felt understood by you as a physician?; complaints taken seriously: do you think your patient felt his complaints were taken seriously?; complaints explained: have you explained to your patient in detail where his complaints may be coming from?; patient agreed: do you have the impression that he could agree with these statements?; patient’s ideas addressed: did you address your patient’s ideas about the origin and treatment of the complaints?; doctor and patient mean the same: to what extent did you get the impression that your patient and you meant the same thing when you talked about his complaint and its treatment?; patient’s opinion regarded: did you feel like you included his perspective and opinion when planning treatment?; responsive to emotions: how responsive were you to the patient’s emotional state?; stressors addressed: to what extent did you also ask about stresses, for example, at work or in the family?

The physician-patient relationship was rated just below the threshold for difficult interactions (cut-off ≥30, mean ± SD in the current sample: 28.6 ± 9.6). Overall, 49.1% of physicians reached a score of ≥30 (Figure 2A). The distribution of the individual items is shown in Supplementary Figure S1.

### Questionnaire on treatment satisfaction from the physician’s point of view

Treatment satisfaction was rated by physicians with a median of 4 (range 0 not at all to 5 very, individual items in Figure 2B).

### General self-assessment of physicians

Physicians overall felt confident in the doctor-patient communication (range 1 not at all to 6 very, ratings 5 or 6 in 82.3%), even in difficult conversations (ratings 5 or 6 in 61.9%, Table 2). Moreover, doctors mostly felt confident in diagnosing (ratings 5 or 6 in 58.6%) and treating (ratings 5 or 6 in 50.3%) patients with functional gastrointestinal disorders (Table 2). While doctors reported to take a lot of time for their patients with functional gastrointestinal disorders (ratings 5 or 6 in 54.8%), only 33.1% rated themselves (ratings 5 or 6) as having enough time for these patients (Table 2). Lastly, only 5.6% (ratings 5 or 6) felt sufficiently compensated for communicating with patients with functional gastrointestinal disorders (Table 2).

**Table 2:** General self-assessment of physicians (*n* = 520).

Question	Rating in % (*n*)					
	Not at all very					
	1	2	3	4	5	6
I feel confident in doctor-patient communication	2.2 (11)		15.2 (79)		82.3 (428)	
I feel confident in difficult patient conversations	3.1 (16)		34.6 (180)		61.9 (322)	
I feel confident in diagnosing patients with functional digestive disorders	3.7 (19)		37.1 (193)		58.6 (305)	
I sometimes prescribe additional diagnostics for patients with functional digestive disorders if they insist on it	6.2 (32)		40.0 (208)		53.5 (278)	
I feel confident in treating patients with functional digestive disorders	3.5 (18)		45.4 (236)		50.3 (262)	
I feel overwhelmed in communications with patients with functional digestive disorders	70.5 (367)		23.1 (120)		5.8 (30)	
I feel uncomfortable in conversations with patients with functional digestive disorders	63.8 (332)		26.5 (138)		9.0 (47)	
I feel annoyed in conversations with patients with functional digestive disorders	54.6 (284)		33.4 (174)		11.5 (60)	
I take a lot of time for conversations with patients with functional digestive disorders	5.0 (26)		39.3 (204)		54.8 (285)	
I have sufficient time for communications with patients with functional digestive disorders	26.2 (136)		40.4 (210)		33.1 (172)	
I am sufficiently compensated for conversations with patients with functional digestive disorders	72.9 (379)		20.4 (106)		5.6 (29)	

If percentage values do not add up to 100%, values were missing from single physicians (max. *n* = 6) for this item.

Physicians largely (ratings 5 or 6) agreed on the statement that the patient feeling satisfied (72.9%) or the doctor feeling satisfied afterwards (66.3%) is an indicator of a successful doctor-patient conversation (Supplementary Table S2). Also, the point that the patient’s wishes coincided with the doctor’s recommendations was rated as an indicator of a successful conversation by 51.5% of physicians (Supplementary Table S2).

## Discussion

Patients and physicians show high agreement on the main hypotheses explaining the origin of their functional gastrointestinal disorder, although, as expected, patients more often suspect somatic causes, whereas physicians recognize the effects of stress-related burdens, which is also addressed in the interviews. This likely leads to the high treatment satisfaction perceived by physicians. Despite these encouraging data and although an experienced collective of primary care physicians was studied (64.7% ≥50 years old, 48.3% ≥20 years of medical practice) also reflected in consistently high ratings regarding confidence in even difficult doctor-patient conversations, the difficulty of the physician-patient relationship is rated just below the threshold and almost half of the physicians reached the cutoff score of 30 indicating a difficult doctor-patient relationship.

The experienced collective of physicians included here likely reflects the sociodemographic situation in Germany (and other Western society countries) right now. Although this collective likely is skilled in communication, data—besides the self-reported data from the current study—are largely lacking. On the patient side functional and or somatoform symptoms more often lead to difficult interactions than “somatic” symptoms (7). Other studies identified mental health as strong predictor for a perceived difficulty of the doctor-patient interaction (8), especially patients with multisomatoform disorders had an odds ratio of 12.3 for being perceived as difficult (9). Interestingly, one study showed that higher frustration with the patient (reflecting difficult doctor-patient relationships) was associated with a physician’s age <40 years (10). This is in line with the current results, and the higher age of the current physician sample may contribute to the subthreshold scoring regarding perceived difficulty of the doctor-patient interaction.

Lastly, the perceived difficulty in the doctor-patient relationship may also derive from the perception of the patient as being time-consuming. Although doctors reported to take enough time for talking to their patients with functional gastrointestinal disorders, only one third strongly agreed to have enough time for these patients and only 5% felt sufficiently compensated for these talks.

The German medical training has been undergoing continuous reformatory and innovative restructuring since the 1990s. Studying medicine takes 6 years consisting of theoretical and practical trainings. Thereafter the licensed physicians start a specialty program of their choice (e.g., internal medicine, vascular surgery, psychiatry, psychosomatic medicine, family medicine, etc.). The specialty training takes another 3–5 years. Contents and a basic understanding of psychosomatic medicine, however, are needed in every specialty. Good doctor-patient-communication positively influences health indicators, patient care and quality of life in patients with end-stage diseases (11, 12). Based on studies, the development and assessment of communication-related competencies during medical education should employ a uniform, integrated and longitudinal curriculum (13, 14). The first medical universities in Germany (e.g., Charité Berlin) have started to include professional communication trainings in 2000. Since then, they have been a substantial part of the medical training with different courses throughout the 6 years of studies. Since 1987, psychosocial services have been a part of the primary care setting in Germany (15). This has been included in the medical education regulations in 1992 and enforced in 1998 (16). However, structured 40 h trainings are only mandatory for general practitioners and in obstetrics/gynecology. Although all other specialties explicitly demand these skills in their curricula they neither teach them in a structured manner nor do they state how they should be acquired.

Furthermore, in a recent multi-center digital survey at five universities, senior medical students stated highest competence for arterial hypertension and lowest for IBS (17). These findings were identical for all institutions irrespective of their curricular model and demographic parameters. 72% of students found that neurogastroenterological diseases should be highlighted more prominently in the curriculum (17). This study shows that some diseases and syndromes are neglected during the training which may lead to missing competence. Taken these facts together, there is a gap between Wants and Haves that must be overcome in Germany (and probably other Western society countries) to meet the needs of patients with functional disorders but also to offer all patients a high-quality doctor-patient communication.

Despite of the strength of the study presenting a large dataset with over 500 physicians and more than 5,000 patients participating, also limitations have to be kept in mind. While only age, duration of symptoms and impairment due to complaints were assessed on the patient side, also other sociodemographic characteristics, not assessed in the current study, such as education, marital status, etc. are likely to play a role in disease coping and therefore might impact on patient-doctor communication. Also, the naturalistic design has two sides. While it is a strength of the study that an unselected sample of physician-patient communications was assessed for patients with a broad spectrum of functional gastrointestinal disorders (long or short duration of symptoms, symptom severity, etc.), this heterogeneity might also likely impact on the perceived communication. Moreover, the current study only considered the physician point of view including the wishes of the patients suspected by the physicians. In addition, although physicians were asked to rate the doctor-patient communication for the next 10 patients with FGID consecutively, this was not ultimately controlled for and therefore a selection bias cannot be completely excluded. Lastly, all data were self-reported bearing a risk of bias.

Based on these findings, (in-depth) education of physicians on functional gastrointestinal disorders (wished for by 64.0% of participating physicians, data not shown), training in physician-patient communication (and/or in primary psychosomatic care, wished for by 42.5% of participating physicians, data not shown) and last but not least a general improvement in the reimbursement of “talking medicine” (wished for by 87.9% of participating physicians, data not shown) could help to (further) increase satisfaction of physicians and patients (18). Moreover, this will also beneficially affect patient adherence (19). Finally, this could reduce the use of expensive, potentially invasive diagnostics, including surgeries, commonly seen in patients with functional gastrointestinal disorders (20).

## Data availability statement

The datasets presented in this article are not readily available because raw data cannot be shared both due to ethical reasons and to data protection laws. All relevant data are within the publication. Requests to access the datasets should be directed to the corresponding author.

## Author contributions

UP, PB, and AS planned the study. AS performed the study. MG-S, UP, PB, SZ, and AS critically evaluated and discussed the data. MG-S and AS wrote the first draft of the paper. All authors contributed to the article and approved the submitted version.

## Funding

This research and its publication were funded by Dr. Willmar Schwabe GmbH & Co. KG, Karlsruhe, Germany. The scientific representatives of Dr. Willmar Schwabe GmbH & Co. KG were used to contact primary care physicians and to advertise the survey. The sponsor was not involved in the study design, collection, analysis, interpretation of data, the writing of this article, or the decision to submit it for publication. The final decision on content was retained by the authors.

## Conflict of interest

MG-S was employed by Helios Klinik Rottweil and received payments for scientific lectures from Dr. Falk Pharma, Dr. Willmar Schwabe GmbH & Co. KG, Medical Tribune, Medice, Microbiotica and Yakult, as well as fees for consulting services from Medice and Yakult. UP and PB are employees of Dr. Willmar Schwabe GmbH & Co. KG, Karlsruhe, Germany. The study was not related to any product and is free of commercial interests. SZ receives loyalties from Frontiers and Thieme Verlag. AS worked as a consultant for a & r Berlin, Boehringer-Ingelheim and Takeda and received payments for scientific presentations from Bayer, Dr. Willmar Schwabe GmbH & Co. KG., Medice, Medical Tribune and Microbiotica.

## Publisher’s note

All claims expressed in this article are solely those of the authors and do not necessarily represent those of their affiliated organizations, or those of the publisher, the editors and the reviewers. Any product that may be evaluated in this article, or claim that may be made by its manufacturer, is not guaranteed or endorsed by the publisher.
