# Supplementary material for: Patients with functional gastrointestinal disorders—importance of communication between physician and patient assessed in a cross-sectional cohort study
Source: Front Psychiatry. 2023 Aug 31;14:1252268. doi: 10.3389/fpsyt.2023.1252268 (PMC10501798; doi:10.3389/fpsyt.2023.1252268)
Supplement: Supplementary file 1 [file Table_1.docx]

**Supplemental TABLE 1.** Characterization of participating patients (n=5,354)

| **Item** | **Frequency (%)** |
| --- | --- |
| Age (years)  ≤ 17  18-29  30-39  40-49  50-59  60-69  ≥ 70  Missing data | f: 0.1, m 0.1  f: 20.1, m 23.8  f: 17.2, m 17.4  f: 16.8, m 16.1  f: 17.2, m 17.1  f: 14.1, m 12.0  f: 12.2, m 10.2  f: 2.4, m 3.4 |
| Duration of symptoms  < 4 weeks  4 weeks - < 3 months  3 months - < 6 months  6 months - < 1 year  1 year - < 5 years  ≥ 5 years  Missing data | 19.3  21.6  19.2  12.4  16.7  10.2  0.5 |
| Impairment due to complaints (1 none to 6 maximum)  1  2  3  4  5  6  Missing data | 0.7  5.3  16.9  29.2  33.8  13.5  0.6 |

Abbreviations: f, female; m, male.

**Supplemental TABLE 2.** Recognition of a successful doctor-patient conversation by the physicians (n=520)

| **Question** | **Rating in % (n)** | | | | | |
| --- | --- | --- | --- | --- | --- | --- |
|  | Not at all Very | | | | | |
|  | 1 | 2 | 3 | 4 | 5 | 6 |
| I have the feeling that my patient is satisfied afterwards. | 1.6  (8) | | 25.0  (130) | | 72.9  (379) | |
| I am satisfied afterwards | 2.7  (14) | | 30.4  (158) | | 66.3  (345) | |
| The patient's wishes coincided with my recommendations. | 6.0  (31) | | 41.9  (218) | | 51.7  (269) | |
| I have enough time for the patient. | 15.0  (78) | | 34.6  (180) | | 50.0  (260) | |

If percentage values do not add up to 100%, values were missing from single physicians for this item.
